# A Guide to the Practical Study of Diseases of the Eye, with an Outline of Their Medical and Operative Treatment

**Published:** 1860-06

**Authors:** 


*A Guide to the Practical Study of Diseases of the Eye, with an Outline of their Medical and Operative Treatment.* By James Dixon, F.R.C.S., Surgeon to the Royal London Ophthalmic Hospital, Moorfield; formerly Assistant-Surgeon to St. Thomas' Hospital. From the Second London Edition. 12mo, muslin, pp. 425. Philadelphia: Lindsay & Blakiston.

This volume has already been five years before the profession, and will not require an analysis at our hands. The treatment of eye diseases has become somewhat of a specialty, especially operative treatment. It is, however, none the less important that every physician acquaint himself as thoroughly in regard to such diseases as any other. It is not every physician who will be called upon to perform the most difficult and delicate operations upon the eye; but he should be prepared to give judicious advice, and acquaint his unfortunate patients of the full resources of our art. Diseases requiring operative treatment are much less common than those demanding medical aid; and however thorough may be the physician's knowledge in regard to eye diseases, that knowledge will find frequent requisition in the discharge of his every-day duties. There are but few, if any, diseases under which the sufferer is more impatient, or more thankful for relief.

The work before us is much less voluminous than Lawrence's or Mackenzie's upon the eye, and on this account, is preferable. In this age of many books, great voluminousness is a great objection, especially in a text-book for the busy practitioner. Dixon's work has another and still more important advantage over the encyclopædial works just referred to: It is of more recent authorship and issue, and thoroughly posts the student in all the modern improvements in diagnosis and treatment of eye diseases. To the general practitioner we recommend the work before us, with pleasure, confident that it is, perhaps, the best work for his use on the subjects upon which it treats, to be found in the English language. This second edition is somewhat enlarged from the former edition, and several of the chapters have been rearranged and rewritten. Should another edition be called for, we would suggest one improvement that would greatly facilitate the convenience of the work for reference. The book is without an *index;* the addition of one, carefully prepared, would greatly enhance its value. A *table of contents*, however copious, will not supersede the necessity of an alphabetically arranged index. The mechanical execution of the work is unexceptionable. O. C. G.

